# Peak frequency of the sensorimotor mu rhythm varies with autism-spectrum traits

**DOI:** 10.3389/fnins.2022.950539

**Published:** 2022-08-05

**Authors:** Caroline C. Strang, Alison Harris, Eric J. Moody, Catherine L. Reed

**Affiliations:** ^1^Department of Psychology, Scripps College, Claremont, CA, United States; ^2^Department of Psychological Science, Claremont McKenna College, Claremont, CA, United States; ^3^Wyoming Institute for Disabilities (WIND), University of Wyoming, Laramie, WY, United States

**Keywords:** mu suppression, peak alpha frequency, individual alpha frequency (IAF), action execution (AE), action observation (AO), mu rhythm, autism spectrum traits

## Abstract

Autism spectrum disorder (ASD) is a neurodevelopmental syndrome characterized by impairments in social perception and communication. Growing evidence suggests that the relationship between deficits in social perception and ASD may extend into the neurotypical population. In electroencephalography (EEG), high autism-spectrum traits in both ASD and neurotypical samples are associated with changes to the mu rhythm, an alpha-band (8–12 Hz) oscillation measured over sensorimotor cortex which typically shows reductions in spectral power during both one’s own movements and observation of others’ actions. This mu suppression is thought to reflect integration of perceptual and motor representations for understanding of others’ mental states, which may be disrupted in individuals with autism-spectrum traits. However, because spectral power is usually quantified at the group level, it has limited usefulness for characterizing individual variation in the mu rhythm, particularly with respect to autism-spectrum traits. Instead, individual peak frequency may provide a better measure of mu rhythm variability across participants. Previous developmental studies have linked ASD to slowing of individual peak frequency in the alpha band, or peak alpha frequency (PAF), predominantly associated with selective attention. Yet individual variability in the peak mu frequency (PMF) remains largely unexplored, particularly with respect to autism-spectrum traits. Here we quantified peak frequency of occipitoparietal alpha and sensorimotor mu rhythms across neurotypical individuals as a function of autism-spectrum traits. High-density 128-channel EEG data were collected from 60 participants while they completed two tasks previously reported to reliably index the sensorimotor mu rhythm: motor execution (bimanual finger tapping) and action observation (viewing of whole-body human movements). We found that individual measurement in the peak oscillatory frequency of the mu rhythm was highly reliable within participants, was not driven by resting vs. task states, and showed good correlation across action execution and observation tasks. Within our neurotypical sample, higher autism-spectrum traits were associated with slowing of the PMF, as predicted. This effect was not likely explained by volume conduction of the occipitoparietal PAF associated with attention. Together, these data support individual peak oscillatory alpha-band frequency as a correlate of autism-spectrum traits, warranting further research with larger samples and clinical populations.

## Introduction

Autism spectrum disorder (ASD) is a neurodevelopmental syndrome associated with impairments in social perception and communication. Growing evidence suggests that deficits in the processing of social information associated with ASD may fall on a continuum extending into the neurotypical population (Constantino and Todd, 2003; Skuse et al., 2005). Using self-report questionnaires such as the Autism-Spectrum Quotient, or AQ (Baron-Cohen et al., 2001), studies of neurotypical samples have found that higher endorsement of autism-spectrum traits is associated with impairments in social judgments (Ingersoll, 2010; Poljac et al., 2013; Becker et al., 2021), as well as changes to the structure and function of the superior temporal sulcus, a brain region linked to social perception (von dem Hagen et al., 2011; Nummenmaa et al., 2012).

In electroencephalography (EEG), differential effects of autism-spectrum traits have been observed for the mu rhythm, an alpha-band (8–12 Hz) neural oscillation measured over sensorimotor cortex. Mu spectral power is typically reduced during both one’s own actions and observation of others’ movements, suggesting it plays a role in integration of perceptual and motor representations for understanding of others’ mental states (for a review, see Fox et al., 2016). Findings of decreased mu suppression both for individuals with ASD (Oberman et al., 2005; Oberman and Ramachandran, 2007) and neurotypical individuals with higher autism-spectrum traits (Siqi-Liu et al., 2018) have led researchers to propose that autism-spectrum traits may be linked to disruptions of sensorimotor action simulation, the idea that we understand the intentions of others through internal reproduction of their movements with our own sensorimotor circuits (Barsalou, 2008; Reed and McIntosh, 2008; Wood et al., 2016; Moody et al., 2018). Notably, autism-spectrum traits have also been associated with an increased prevalence of dyspraxia, or impairment in planning and executing movements, both in neurotypical and ASD samples (Cassidy et al., 2016). Thus, the integration of perceptual and motor representations may be disrupted in individuals with higher autism-spectrum traits.

However, because spectral power is usually quantified at the group level, this measure has limited usefulness for characterizing individual variation in the sensorimotor mu rhythm. For example, despite the existence of a significant group-level difference in spectral power of the mu rhythm between high- and low-AQ neurotypical participants (Siqi-Liu et al., 2018), a correlation analysis on the same data was not significant (*p* > 0.2; Harris, 2018 unpublished data). Instead, individual peak frequency may provide a better measure of variability in the mu oscillation across participants, particularly with respect to autism-spectrum traits. An individual or peak frequency is commonly defined as the most prominent spectral peak within a specific frequency band for a given participant (Corcoran et al., 2018). Previous research has examined the peak alpha frequency (PAF), commonly defined as an individual’s most prominent spectral peak in the alpha band between 7 and 13 Hz (Posthuma et al., 2001; Grandy et al., 2013). PAF appears to be a stable and heritable trait in adults, and has been linked to global cognitive function (Klimesch et al., 1990; Anokhin and Vogel, 1996; Clark et al., 2004; c.f., Grandy et al., 2013). Moreover, developmental data suggest that PAF is slower in individuals with ASD compared to neurotypical controls (Edgar et al., 2015, 2019; Dickinson et al., 2018), making it a potential biomarker for ASD (Dickinson et al., 2018). These results support the idea that individual peak frequency of alpha-band EEG oscillations could potentially index individual variation in autism-spectrum traits even within the neurotypical population.

Yet, in contrast to the robust literature on individual variation in PAF, individual variability in the peak frequency of the mu rhythm, or peak mu frequency (PMF), remains largely unexplored. The few studies to quantify PMF have largely focused on its development across the lifespan (Berchicci et al., 2011; Thorpe et al., 2016). In these experiments, mu rhythms during motor execution vs. resting baseline were compared in three groups: infants, children, and adults. In line with previous research on the developmental trajectory of PMF in infants (Stroganova et al., 1999), these studies reported an increase in individual PMF with age, with an average for adults between 10 and 12 Hz.

These results suggest that variability in the peak frequency of the sensorimotor mu rhythm may be measurable in adults, similar to previous reports for PAF (Posthuma et al., 2001; Grandy et al., 2013). However, one caveat in comparing existing findings regarding PAF and PMF is that most studies of PAF have focused on global resting-state oscillations rather than task-evoked activity. Although recent evidence suggests that the same brain networks underlie both types of oscillations (Mierau et al., 2017), the relation of resting-state and task-evoked alpha-band rhythms has been debated. Therefore, the extent to which resting-state and task-evoked peak frequencies measured within the same participants are correlated remains unclear.

A further question is that of how peak oscillatory frequencies are correlated across different tasks. Although the mu rhythm was originally defined by comparing motor execution and rest, suppression of spectral mu-band power at the group level has also been reported during observation of others’ actions. These results have led researchers to link mu suppression to various aspects of social perception, including observations of other’s goal-directed movements (Muthukumaraswamy et al., 2004), social context in hand-game playing (Perry et al., 2011), and classification of facial emotions (Moore and Franz, 2017). Such results have been cited as evidence for action simulation theory, the idea that others’ intentions are understood through internal sensorimotor simulation of their external actions. If both action execution (AE) and action observation (AO) depend on the same neural circuits, the PMF calculated for each participant individually should be highly correlated across AE and AO tasks.

Finally, previous research on individual peak alpha-band frequencies has not definitively distinguished between occipitoparietal alpha oscillations and the sensorimotor mu rhythm. The spectral power of the more posterior alpha rhythm is known to be suppressed during attention relative to baseline (Foxe and Snyder, 2011; Payne and Sekuler, 2014), and has previously been identified as a potential confound in many studies of mu suppression (Hobson and Bishop, 2017). For example, a topographic analysis of PMF found that mu suppression associated with motor execution extended into parietal regions, perhaps reflecting contributions from the posterior alpha rhythm (Thorpe et al., 2016). This raises the question of whether these two measures and their associated cognitive processes can be separated. Measurement of PMF could thus potentially be conflated with global alpha-band oscillations, particularly when computed with resting-state data that does not strongly control for attention.

**FIGURE 1:**
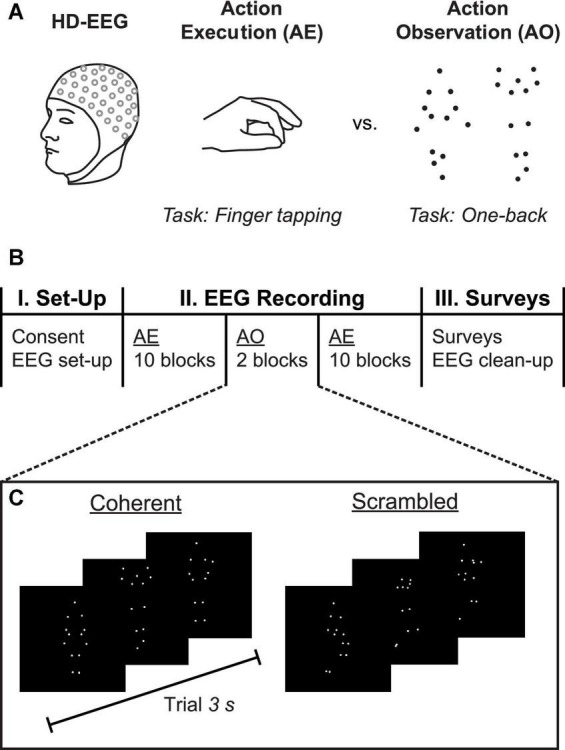
Experimental methods. **(A)** High-density 128-channel EEG was collected while participants completed separate blocks of two different tasks: action execution (AE), in which the participant was instructed to alternate between resting and tapping the index and middle fingers of both hands against the respective thumbs; and, action observation (AO), in which the participant observed point-light display (PLD) animations of whole-body human movements while continuously monitoring for immediate repetitions (one-back task). **(B)** Timeline of experiment. Participants completed 10 blocks of AE, followed by two blocks of AO, and another 10 blocks of AE. Following the EEG recording, participants filled out surveys including the autism-spectrum quotient (AQ) questionnaire. **(C)** Sample trials from the AO task, which included one block each of *Coherent* biologically plausible PLDs and *Scrambled* stimuli in which the starting locations of the dots were randomly displaced. Each PLD animation had a duration of 3 s.

To address these questions, the present study explored individual variation in PMF within a sample of neurotypical young adults. In this experiment, participants alternated between blocks of performing a simple finger-tapping motor task and viewing point-light displays (PLDs) of people enacting whole-body movements (Figure 1). First, we quantified individual variation in the sensorimotor mu rhythm, and measured the correlation between PMF during resting-state and task-evoked data. Next, we directly tested whether peak oscillatory frequency of the mu rhythm is correlated across different tasks by comparing PMF during the AE and AO conditions. Finally, we examined whether PMF correlated with self-reported autism-spectrum traits, and how this measure compared to the PAF. Based on previous research, we predicted that PMF would be slower in individuals with higher autism-spectrum traits, as indicated by higher AQ.

## Materials and methods

### Participants

Undergraduate students (*N* = 70; ages 18–23, 45 females) were recruited from the local college community. Ten participants were excluded, due to differences in the pilot version of the experiment (*n* = 3), recording issues or excessive noise in the EEG data (*n* = 4), and poor behavioral performance (*n* = 3). Compensation took the form of partial course credit or cash payment. All procedures were approved by the college’s Institutional Review Board, and participants provided informed consent in writing prior to the start of the experiment.

### Stimuli

The AO task employed stimuli from a previous study (Siqi-Liu et al., 2018), consisting of point-light display videos displaying actors performing whole-body actions (Figure 1). PLDs preserve biological motion information while discarding irrelevant visual body shape cues that can bias the neural response. The PLDs were 3 s video clips of whole-body actions used in a previous study (Atkinson et al., 2012), selected from a larger validated database (Atkinson et al., 2004). The stimuli included bodily expressions of emotion (happiness, anger, sadness) and affectively neutral but meaningful actions (touching toes, marching in place, hopping on one foot) produced by both male and female actors. To control for low-level visual input, we also presented “scrambled” versions of the same stimuli in which the starting positions of the dots were randomized within the bounds of the original viewing frame (Atkinson et al., 2012). These scrambled PLDs contained the same low-level motion cues as the coherent PLDs, but the dots no longer formed biologically plausible actions. The PLDs included six different performances of each emotion (18 different videos total) and two different performances of each neutral action (6 different videos total), along with their corresponding scrambled versions. Thus, there were 24 PLDs per condition, for a total of 48 distinct PLDs.

### Procedure

High-density 128-channel EEG was used to record the participant’s brain activity while he or she engaged in a set of tasks designed to elicit mu suppression: AE and AO (Figure 1A). The timeline of the experiment is shown in Figure 1B. Following application of the EEG head cap, each participant completed 2 runs of the AE task (approximately 5–6 min each), interleaved with the AO task (approximately 20–25 min).

The AE task consisted of 2 runs of 10 blocks each, in which participants were instructed to alternate rest with bimanual finger tapping. The rest condition was cued with a red square on the computer screen, during which participants were instructed to remain still. When the red square was replaced by a green dot, participants tapped their thumbs against their middle and index fingers repeatedly. Each block of rest and finger tapping lasted 16 s each, for a total of 32 s per block. The participants were instructed to place their hands in their laps under a table such that the finger tapping action could not be observed, and to maintain visual fixation on the screen.

For the AO task, we used the same parameters described in Siqi-Liu et al. (2018). Stimuli were presented in MATLAB using the Psychophysics Toolbox (Brainard, 1997; Kleiner et al., 2007). Participants viewed a series of PLDs displaying actors performing whole-body movements that were emotional or affectively neutral. Each PLD played for 3 s, freezing on the last frame for the duration of the 2-s intertrial interval (ITI). To ensure attentive processing of all stimuli, participants were asked to complete a continuous “one-back” monitoring task, in which they determined whether the current stimulus exactly matched the previous stimulus (i.e., whether the specific dot configuration and motion for each video was presented twice in a row). If the participant determined the PLD most recently viewed was a consecutive repeat of the immediately preceding PLD, they responded *via* a button press within the 2-s ITI. Participants received 10 practice trials (5 coherent and 5 scrambled, 1 one-back trial each) before beginning experimental trials for the AO task.

Point-light displays were presented in two separate blocks of Coherent (biologically plausible actions) and Scrambled (biologically meaningless, low-level visual motion control) conditions (Figure 1C). Block order was counterbalanced across participants. Each of 18 emotional PLDs and 6 neutral PLDs were presented 3 and 9 times, respectively, resulting in 108 trials per block. For both blocks, 12 trials were randomly selected to be followed by one-back repetitions, generating 240 trials total (216 stimulus presentations plus 24 one-back trials). Within each block, stimuli were pseudo-randomly interleaved to ensure the correct number of target “one-back” trials. Participants with poor behavioral performance (d-prime <1) in either of the AO conditions were excluded from further analysis.

Following the EEG tasks, participants completed a two-question, multiple-choice manipulation check to assess their ability to identify which actions and emotions they had seen, as well as a digital version of the AQ. The AQ is a self-administered questionnaire designed to measure autistic tendencies in the domains of social skills, attention switching, attention to detail, communication, and imagination (Baron-Cohen et al., 2001). Although it is not a diagnostic instrument, the AQ has been used in many studies as a measure of self-assessed autistic trait expression (e.g., Poljac et al., 2013).

### Electroencephalography data acquisition and analysis

Electroencephalography data was collected using the BioSemi ActiveTwo EEG system (BioSemi B.V., Amsterdam, Netherlands) with 128 active electrodes inserted in fitted head caps. Two additional electrodes were bilaterally placed on the mastoids to serve as reference channels. EEG signals were digitized continuously at a sampling rate of 512 Hz with a hardware low-pass at one-fifth of the sampling rate. Prior to data collection, offsets were adjusted to fall between −30 and 30 mV with no obvious slow drifts in the online data measurement.

Data preprocessing was performed offline in MATLAB (The MathWorks Inc., Natick, MA, United States) using the EEGLAB toolbox (Delorme and Makeig, 2004). The preprocessing stream included linear detrending and a high-pass filter of 0.5 Hz to remove slow voltage drifts. Data epochs were extracted for a time window of −9 to −1 s pre-action cue and 1 to 9 s post-action cue in the AE task, corresponding to Rest and Action periods, and from −400 ms pre-stimulus to 3,200 ms post-stimulus onset in the AO task. For the AO task, data were resampled to 500 Hz to enable comparison to other datasets from our lab; estimates of PMF did not significantly differ between resampled and original data. Data epochs in the AO task were also baseline-corrected to the pre-stimulus period (−400 to 0 ms). To exclude potential motor preparatory activity, all one-back trials and other trials where participants made a motor response were removed from data analyses. Artifactual signals in the EEG data (oculomotor, muscle, electrical, and sensor noise) were identified and removed from the remaining trials for each participant using independent component analysis *via* second-order blind identification (Belouchrani et al., 1997; Tang et al., 2005). Selections were cross-checked with automated artifact classifications from the MARA (Winkler et al., 2011) and ICLabel (Pion-Tonachini et al., 2019) plugins for EEGLAB, and the task-related components were then projected back onto the scalp (Jung et al., 2000).

To identify sensors showing a statistically significant sensorimotor mu suppression effect at the group level, we performed a spectral decomposition using the Field-Trip toolbox for time-frequency analysis (Oostenveld et al., 2011) in MATLAB. For each participant’s data from the AE task, we separately computed power spectra in the Action (finger tapping) and Rest (baseline) conditions. Power spectra were computed for frequencies from 1 to 30 Hz using the fast Fourier transform with multi-taper method, averaging across both trials and the entire 8-s epoch. Frequency data was log_10_ transformed to normalize the frequency distribution. Preliminary inspection of the data revealed that the mu suppression effect was localized to approximately 10 to 14 Hz, so this time window was used for subsequent statistical analysis. Sensors of interest (SOIs) within the 10–14 Hz frequency band were identified *via* a dependent sample two-tailed *t*-test with a non-parametric cluster-based Monte Carlo permutation test (1,000 repetitions) to correct for multiple comparisons, with significance defined by an overall threshold of *p* = 0.05 (*p* = 0.025 at each tail). SOIs for the mu rhythm were defined as the cluster of sensors showing a significant reduction in power during Action relative to Rest (Action < Rest) conditions, whereas SOIs for the occipitoparietal alpha rhythm were defined as those showing a significant positive effect (Action > Rest).

Peak mu frequency for AE (rest and finger-tapping conditions) and AO (coherent and scrambled conditions) were calculated separately for each individual using an automated procedure created by Corcoran et al. (2018). The method uses a Savitzky-Golay filter to smooth power spectra and detect spectral peaks. For our analysis, we employed the default settings of the automated program from Corcoran et al. (2018), which included band-pass filtering from 1 to 40 Hz and peak search window ranging from 7 to 13 Hz. However, to ensure that we had the highest likelihood of identifying a peak frequency in the alpha band for each individual participant, we set the minimum number of channel estimates required for cross-channel averages to 1 (rather than 2 in the original program). For current source density (CSD) analysis, the data from EEGLAB were first converted using the CSD Toolbox (Kayser and Tenke, 2006) for MATLAB.

## Results

### Calculation of peak mu frequency

**FIGURE 2:**
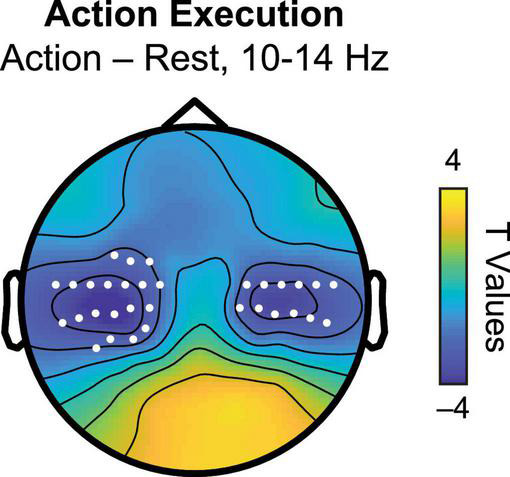
Scalp topography of *t* statistics for action execution (Action–Rest) between 10 and 14 Hz. White markers indicate sensors of interest (SOIs) associated with significant mu suppression in this frequency band used as sensors of interest in the PMF analysis.

For our first analysis, we sought to quantify individual variation in the PMF during our two tasks. To ensure that we were using appropriate sensor locations associated with the sensorimotor mu rhythm, we first identified electrodes that showed a significant reduction in spectral power during the AE task at the group level. Comparing the Action and Rest conditions, we observed significant decreases in power from approximately 10 to 14 Hz over central electrode sites, consistent with a substantial literature on sensorimotor mu suppression (Fox et al., 2016). Figure 2 displays the scalp topography of the mu suppression effect derived from the group comparison, with sensors reaching significance at *p* < 0.05 (cluster-corrected permutation test) indicated in white. For the main analysis, data for each task and condition in each participant were then extracted from these SOIs.

Peak frequency is typically identified as the most prominent spectral peak based on visual inspection of the power spectrum (Corcoran et al., 2018). However, the existence of either split peaks (Chiang et al., 2011) or no peaks at all within the frequency band is not uncommon (Corcoran et al., 2018). In our analyses, PMFs for some participants simply could not be determined, whereas others had PMF estimates only for a subset of the conditions. Therefore, participants without the relevant PMF were excluded independently for each analysis, resulting in different sample sizes for each comparison. Analyzing the subset of participants with reliable PMFs across all tasks and conditions (*N* = 41) revealed a similar pattern of significant results.

Since our research question seeks to quantify individual variation in a neural measure, a first question was whether estimation of the PMF is reliable within individual participants. In particular, comparing even and odd trials allows measurement of reliability across trials while accounting for potential confounds over time (e.g., fatigue, practice). Therefore, we computed the correlation of even- and odd-numbered trials from the Coherent biological motion condition in the AO task. In this analysis, PMFs were successfully computed in both even and odd trials for 45 out of 60 participants. We found a large, significant correlation of individual PMF between even and odd AO trials [*r*(43) = 0.94, *p* < 0.001]. To account for the reduction in reliability due to effectively halving the number of trials we further applied the Spearman-Brown correction, yielding an estimated reliability of *r* = 0.97. These results support high internal reliability of the PMF measurement within participants.

### Resting-state vs. task-evoked peak mu frequency

Although we successfully identified individual variation in PMF, one caveat is that task-evoked alpha-band activity may have different properties from the resting-state alpha rhythm characterized in previous studies. Therefore, our next question concerned whether the PMF identified for an individual differed as a function of resting state vs. task demands. We compared the PMF in the AE task during Action (finger-tapping) vs. Rest (baseline). At only 16 s each, rest blocks were shorter than a typical extended resting-state EEG recording, though across 20 blocks of trials they added up to a similar duration (approximately 5 min). Data from 52 participants contributed to this analysis. Strikingly, the PMF identified for each individual during Action vs. Rest was strongly correlated [*r*(50) = 0.8, *p* < 0.001]. Thus, oscillatory alpha-band rhythms over sensorimotor cortex appear similar regardless of whether participants are sitting quietly or actively engaged in finger tapping.

We also tested whether a similar correlation of resting-state and task-evoked peak frequency could be observed for the posterior alpha rhythm. Since the majority of previous studies of PAF in ASD have looked at resting-state data (Edgar et al., 2015, 2019; Dickinson et al., 2018), it is an important question whether these effects generalize across resting states and task demands. Individual PAF was computed for each participant from EEG data extracted at occipitoparietal sensors. Data from 52 participants contributed to this analysis. Again, the correlation of Action and Rest was highly significant [*r*(50) = 0.96, *p* < 0.001]. This suggests that the use of task-evoked rather than resting-state data is unlikely to limit the generalizability of our findings.

### Peak mu frequency across tasks: Action execution vs. action observation

Having demonstrated that sensorimotor PMF is highly similar across resting and task states, we next examined whether the individual PMF varies as a function of task demands. Previous research has shown that decreases in spectral power of the mu rhythm occur both for one’s own motor movements and for observation of others’ actions (Fox et al., 2016). If the mu rhythm across these conditions reflects the same underlying neural circuits, as proposed by action simulation theory, then we would predict that the PMF calculated for each participant individually would be significantly correlated across the two tasks.

**FIGURE 3:**
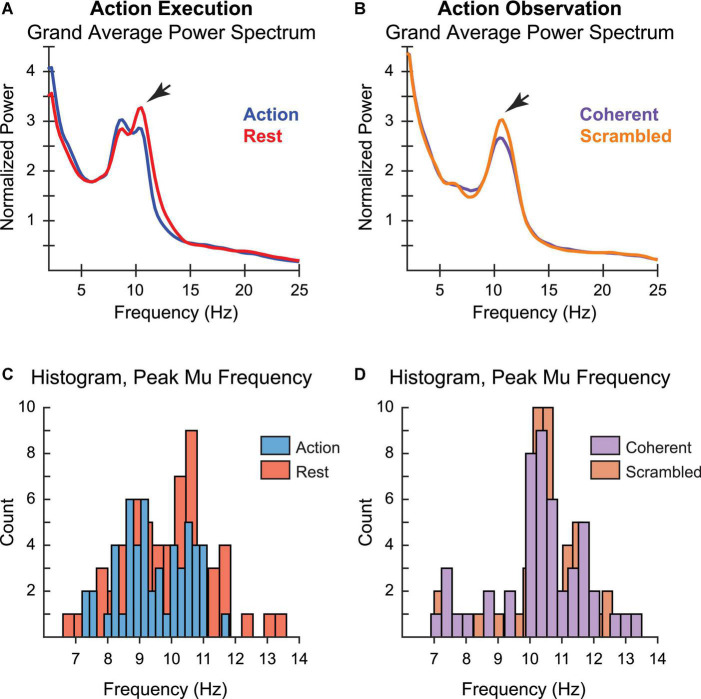
Peak mu frequency for action execution (left) and action observation (right). Grand average power spectrum for **(A)** action execution, action (blue) vs. rest (red), and **(B)** action observation, coherent (purple) vs. scrambled (orange) movement. Histogram of individual measurements of peak mu frequency for **(C)** action execution, action (blue) vs. rest (red), and **(D)** action observation, coherent (purple) vs. scrambled (orange) movement. The varying width of the bars for each condition reflects the number of bins for the histogram, selected automatically *via* algorithm to best cover the range of values while revealing the shape of the underlying distribution.

First, to verify that we had replicated the finding of mu suppression in our AE and AO tasks, we examined spectral power data at the group level. AE was associated with a peak centered between 10 and 11 Hz, though an additional clustering of individual PMF around 9 Hz was also present in the AE condition (Figure 3A). However, only the 10–11 Hz peak showed the predicted pattern of greater suppression during motor execution (Chatrian et al., 1959). Supporting this observation, statistical testing showed that reductions in power during Action relative to Rest achieved significance for individuals with a PMF greater than 10 Hz (*t*(18) = −2.3, *p* = 0.03, [95% CI: −2.16 to −0.1]), but not for those with whose PMF was below 10 Hz (*t*(32) = −1.27, *p* = 0.21, [95% CI: −1.11 to 0.26]). This finding is consistent with other reports that the sensorimotor PMF predominantly reflects spectral power in the upper alpha band (Thorpe et al., 2016). In contrast, during AO we found only a single peak in the 10–11 Hz range (Figure 3B), which was significantly larger during observation of scrambled vs. coherent PLDs (*t*(45) = −2.18, *p* = 0.03, [95% CI: −0.52 to −0.02]), replicating previous reports of mu suppression during observation of PLDs (Ulloa and Pineda, 2007; Siqi-Liu et al., 2018). Further supporting these observations, histograms of the frequency distribution across individuals showed a distribution centered between 10 and 11 Hz for PMF in both AE and AO conditions, but with an additional clustering around 9 Hz in the AE condition (Figures 3C,D).

**FIGURE 4:**
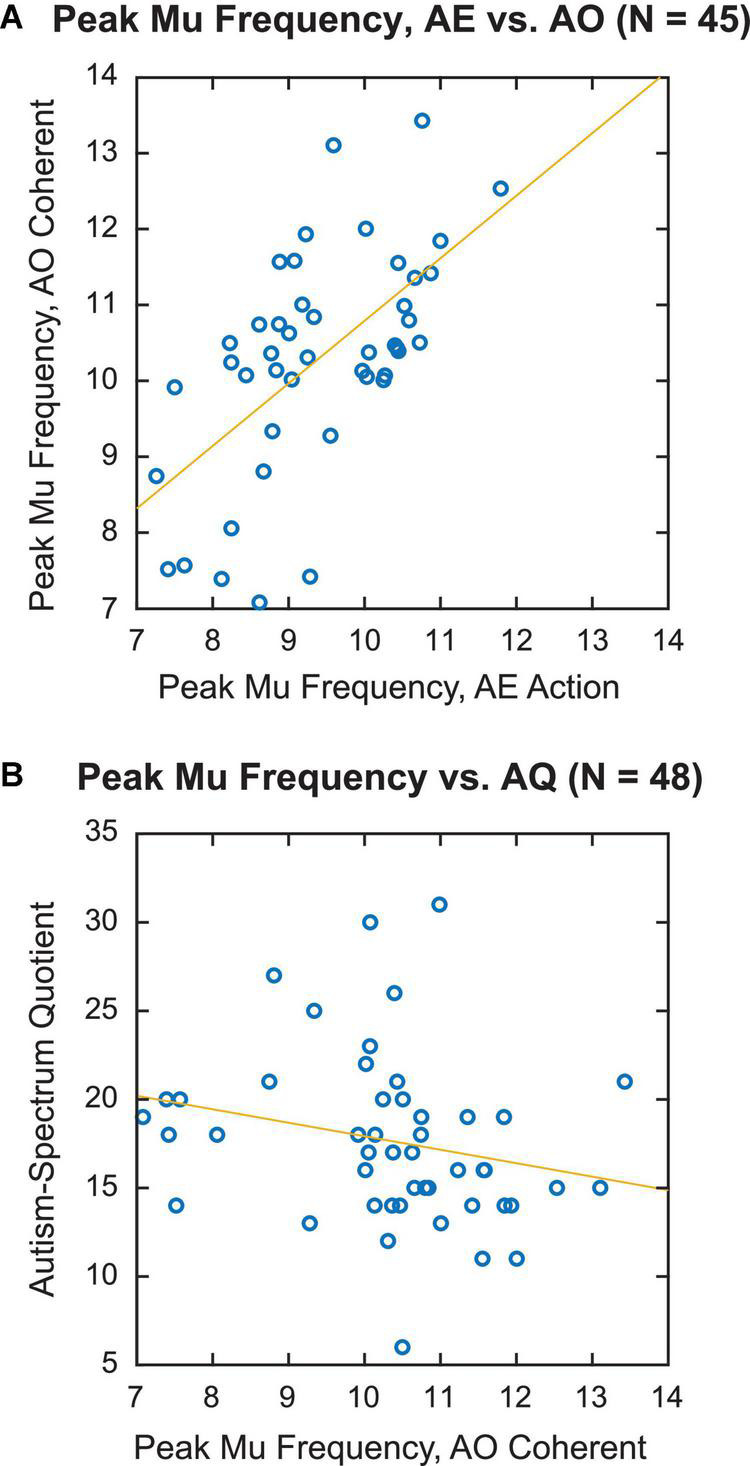
Individual variation in peak mu frequency. **(A)** Correlation of individual peak mu frequency for action execution, action condition vs. action observation, coherent condition. **(B)** Individual peak mu frequency for action observation, coherent condition vs. autism-spectrum quotient (AQ) score.

To determine whether individual sensorimotor PMF was similar across different tasks, we correlated the PMF obtained during the AE and AO tasks across participants. Specifically, we compared Action in the AE task and Coherent PLD in the AO task, since these conditions were associated with the largest reductions in mu power. Figure 4A displays a scatterplot of individual PMF for the 45 participants for whom it could be calculated in both AE and AO tasks. There was a clear positive relationship between PMF in the two conditions, with higher values of PMF during AE associated with higher values of PMF during AO as well. Statistical analysis confirmed a highly significant correlation between PMFs for finger tapping and observing coherent PLDs [*r*(43) = 0.61, *p* < 0.001]. Thus, these results are consistent with the idea of common neural circuits underlying mu rhythms across both motor execution and observation of others’ actions, in line with the predictions of action simulation theory.

### Peak mu frequency as a function of autism-spectrum quotient

In our previous analyses, we verified that there is measurable individual variation in the sensorimotor PMF, that this variability is similar across resting and task-evoked data, and that the PMF is significantly correlated during independent tasks previously associated with mu suppression. Our last question concerned whether individual variation in PMF correlates with differences in self-reported autism-spectrum traits.

For this analysis, we focused on the PMF for the Coherent PLD condition in the AO task, as the AQ indexes social communication skills but not motor capabilities *per se*. Forty-eight participants were included in this sample on the basis of the PMF calculation in this condition. For this sample, the average AQ score was 17.6 (median = 17, SD = 4.82). These values fall within the typical range of social functioning for this measure (i.e., 11–21), as determined from previous studies (Baron-Cohen et al., 2001). One potential issue was that our sample included a relatively high number of females (32 out of 48). Since ASD is more prevalent in males than females, this gender imbalance may limit the distribution of autism-spectrum traits measured in our sample. Indeed, there was a trend for AQ scores to be higher in male (mean = 19.5, SD = 5.89) vs. female (mean = 16.7, SD = 3.98), which approached significance [*t*(46) = −1.94, *p* = 0.06].

Nonetheless, as predicted, we observed a negative correlation between PMF and AQ score within our neurotypical sample, such that participants who scored higher on the AQ had a decreased PMF (Figure 4B). Because initial inspection of the data revealed a data point with high leverage and Cook’s distance scores, we calculated the correlation with Spearman’s rank test, which is more robust against observed outliers. This analysis yielded a significant negative correlation [Spearman’s ρ(46) = −0.34, *p* = 0.017]. Analyzing just the subset of 41 participants with measurable PMF across all conditions confirmed the direction and significance of this effect [Spearman’s ρ(39) = −0.46, *p* = 0.003].

For the sake of comparison, we also computed the correlation of spectral mu power and AQ score in our current dataset for the same frequency band (9 to 12 Hz) and time window (1 to 2 s post-stimulus onset) used our previous analysis (Siqi-Liu et al., 2018). Again, the correlation was not significant (*p* > 0.4), suggesting that group-level differences in spectral power are suboptimal for capturing individual variation in the mu rhythm.

### Peak mu frequency vs. peak alpha frequency

Our finding of a negative correlation between sensorimotor PMF and AQ is broadly consistent with previous reports of slower global peak alpha-band rhythms in ASD (Dickinson et al., 2018; Edgar et al., 2019). However, one potential concern is that our results could simply reflect transmission of the occipitoparietal alpha rhythm *via* volume conduction. Volume conduction of the EEG signal is particularly problematic in recordings of oscillatory activity because different rhythms at the same frequency may be conflated across recording sites (Tenke and Kayser, 2015).

To assess this possibility, we computed the individual PAF across participants from EEG data extracted at occipitoparietal sensors. In fact, the PAF computed at occipitoparietal sensors during observation of Coherent PLDs was highly negatively correlated with AQ [*r*(40) = −0.46, *p* = 0.002]. This extends previous findings of slowed PAF in children with ASD to autism-spectrum traits in the neurotypical adult population. However, it also raises concerns about the contribution of PAF to the measured PMF. Therefore, we also computed the PMF for the current source density (CSD), or surface Laplacian, transformation of the EEG data (Kayser and Tenke, 2006). CSD provides a spatially sharpened signal that is less influenced by volume conduction (Tenke and Kayser, 2012). Accordingly, CSD-transformed signals measured over central sensors should more closely reflect neural generators in sensorimotor cortex (e.g., Burle et al., 2015).

**FIGURE 5:**
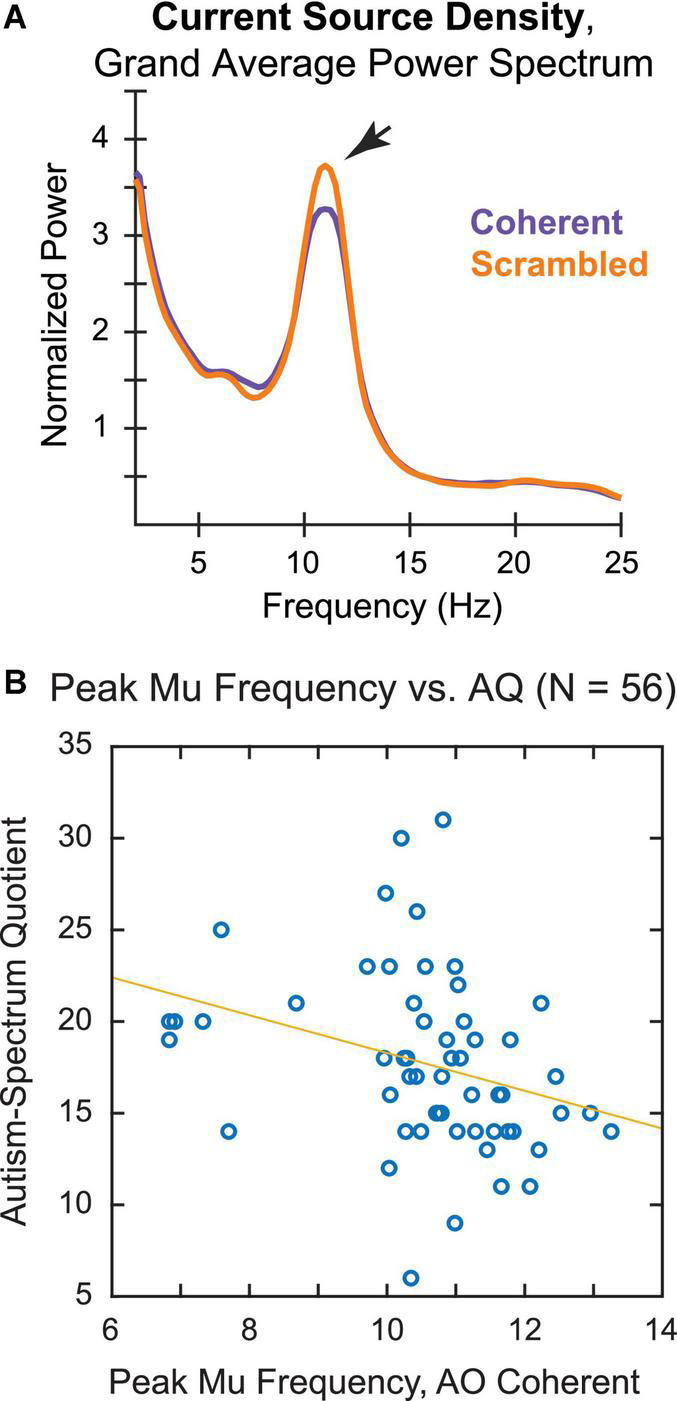
Current source density (CSD) analysis of **(A)** grand average power spectrum and **(B)** correlation of individual PMF during action observation of biologically plausible PLDs with AQ score.

Peak mu frequency calculations for the CSD-transformed data are shown in Figure 5. Similar to the original analysis, the grand average power spectrum revealed a peak between 10 and 11 Hz that was larger for observation of Scrambled, as opposed to Coherent, whole-body PLDs (Figure 5A). Notably, using the CSD-transformed data we were also able to identify an individual PMF in response to the Coherent PLDs in an overwhelming majority of participants, with only four participants excluded from this analysis.

A scatterplot of the PMF during observation of Coherent whole-body PLDs by AQ score again showed a negative relationship between PMF and AQ (Figure 5B), suggesting that neurotypical participants with higher AQ scores tended to have slower mu rhythms. Consistent with this observation, the correlation coefficient was significantly negative [Spearman’s ρ(54) = −0.41, *p* = 0.002]. Whereas the correlation of PMF and AQ remained strong using CSD-transformed data, the correlation between PAF and AQ became weaker following CSD transformation [*r*(48) = −0.28, *p* = 0.051]. Thus, it seems unlikely that the connection between PMF and autistic tendencies in our neurotypical sample can be explained merely by volume conduction of the occipitoparietal alpha rhythm. This further supports the idea that differences in sensorimotor action simulation may be tied to variability in autism-spectrum traits, along with more general differences in global alpha-band oscillations among these individuals.

## Discussion

Although impairments in social perception are a diagnostic hallmark of ASD, converging evidence suggests that differences in social perception indexed by autism-spectrum traits may extend into the neurotypical population. Findings from behavioral (Ingersoll, 2010; Poljac et al., 2013; Becker et al., 2021), neuroimaging (Nummenmaa et al., 2006; von dem Hagen et al., 2011), and EEG (Siqi-Liu et al., 2018) studies suggest that higher endorsement of autism-spectrum traits is associated with alterations of social perception and associated brain structure and function, even within neurotypical samples. In particular, we focused here on the mu rhythm, an alpha-band oscillation previously hypothesized to index sensorimotor simulation of others’ external actions in order to understand their internal states. Differences in mu suppression during AO have previously been described at the group level for neurotypical individuals with high and low autism-spectrum traits (Siqi-Liu et al., 2018). In this study, we extended this research by examining individual variability in the peak frequency of the sensorimotor mu rhythm, measured across neurotypical individuals as a function of autism-spectrum traits.

Previous research has suggested that individual peak frequency of the occipitoparietal alpha rhythm may be a biomarker for cognitive function, with some studies showing slower PAF in individuals with ASD (Edgar et al., 2015; Dickinson et al., 2018). However, comparison of PAF and PMF is complicated by the fact that much of the research on PAF relies on resting-state data, whereas PMF is typically defined by task-evoked activity (Berchicci et al., 2011; Thorpe et al., 2016). Additionally, previous studies of PMF have focused largely on mu rhythms elicited by motor execution, whereas research on social perception suggests that changes in the spectral power of the mu rhythm are also produced by observation of others’ actions. Therefore, we performed a series of analyses to explore individual variation in the PMF during both resting and task states, as well as across AE and AO tasks. Finally, we correlated peak frequency of the mu rhythm with AQ score, to determine whether this measure indexes individual differences in social perception associated with autism-spectrum traits.

We first verified that variability in the PMF was measurable in our sample of neurotypical adults. Consistent with previous descriptions of sensorimotor PMF between 10 and 12 Hz (Berchicci et al., 2011; Thorpe et al., 2016), we observed that the frequency distribution of peak mu was centered between 10 and 11 Hz for both AE and AO tasks. However, we also identified two separate alpha-band peaks at central sensors during AE: one from approximately 8–10 Hz, centered around 9 Hz, and one from 10 to 12 Hz, centered between 10 and 11 Hz. Although some previous research has distinguished between low and high bands of rhythmic mu activity during motor movement and AO (Andrew and Pfurtscheller, 1997; Pfurtscheller et al., 2000; Frenkel-Toledo et al., 2013), recent developmental data suggest that PMF in adults is best fit by a single broad peak (Thorpe et al., 2016). Nonetheless, when Thorpe et al. (2016) compared the upper and lower alpha bands across all age groups, they found that mu suppression effects during AE were concentrated in the upper alpha band. Our findings are consistent with this observation, as only the upper-band PMF in our participants showed a specific decrease in spectral power during action relative to rest.

Yet, it should be noted that there is substantial heterogeneity in the empirical evidence regarding the functional significance of upper and lower sub-bands for the mu rhythm. Whereas some studies report selective mu suppression associated with the upper 10–12 Hz band (Pfurtscheller et al., 2000), other results conversely implicate the lower band from 8 to 10 Hz (Marshall et al., 2009; Frenkel-Toledo et al., 2013). One study specifically investigated mu suppression across lower and upper sub-bands in neurotypical individuals vs. individuals with ASD (Dumas et al., 2014). Both groups displayed similar mu suppression for the lower sub-band while suppression in the higher sub-band was specifically reduced for individuals with ASD, which the authors attribute to differences in inhibitory control rather than sensorimotor function *per se*. However, aside from Thorpe et al. (2016), the majority of these studies have averaged mu spectral power across frequencies within set bands rather than identifying individual peak frequencies. Further research indexing individual PMF may thus potentially provide new insights into the functional relationships between different sub-bands of the mu rhythm.

In addition to quantifying PMF across individuals, we also compared peak frequencies of the mu rhythm within participants as a function of resting state and task demands. We found that individual measurement of the PMF was highly reliable, was not affected differentially by resting vs. task states, and showed good correlation across different tasks. These results support the idea that reductions in the mu rhythm over central electrodes during AE and AO reflect the same underlying neural sources, a fundamental assumption underlying action simulation theory. Our data also join growing evidence that trait variability in the peak frequency of alpha-band activity is directly related to state-dependent activation of neural populations (Mierau et al., 2017). Although much of the data on short-term state-dependent changes has looked at the occipitoparietal alpha rhythm, the sensorimotor mu rhythm could conceivably be investigated in a similar manner. Future studies could further tease apart the trait- and state-dependent elements of the mu rhythm by comparing PMF elicited across a variety of task demands (e.g., increasing physical or cognitive effort), as well as the role of factors such as mood, age, or physical exercise. Inasmuch as PAF and PMF both index individual variation in cognitive processing, these measures also provide complementary information given their association with specific underlying neural circuits for selective attention vs. sensorimotor planning and execution.

For our final analysis, we investigated the relationship between individual variability in the PMF and endorsement of autism-spectrum traits. Neurotypical participants who self-reported higher autism traits tended to display slower PMFs, consistent with previous findings of slowed PAF in ASD samples (Edgar et al., 2015, 2019; Dickinson et al., 2018). These results are all the more striking given that the average score in our neurotypical sample fell within the normal functioning range of the AQ. Indeed, only two participants had AQ scores (30 and 31) that approached the threshold indicative of a high likelihood of diagnosable ASD (AQ = 32). Therefore, any differences in PMF across our sample are unlikely to reflect undiagnosed ASD or other gross impairments in social perception and communication.

However, the AQ is not a clinical tool, and despite its widespread use in neurotypical samples, recent analyses suggest that responses to certain items differ by gender and age (Agelink van Rentergem et al., 2019). In this study, the highly skewed gender ratio was of particular concern, with two-thirds of our sample composed of females. Further work should explore whether the correlation reported here replicates in larger and more diverse neurotypical samples, as well as in individuals with a clinical diagnosis of ASD. Other measures of autism-spectrum traits such as the Broad Autism Phenotype Questionnaire (BAPQ) could also be administered to supplement characterization of autism-spectrum traits in neurotypical samples (Hurley et al., 2007). Finally, the correlation between PMF and other behavioral differences associated with higher autism-spectrum traits could be explored in the future. For example, given the finding of higher levels of dyspraxia in neurotypical individuals high in autism-spectrum traits (Cassidy et al., 2016), it may be of interest to measure individual PMF under conditions of postural instability, which have previously been demonstrated to affect individual PAF (Hulsdunker et al., 2015).

One limitation to the current findings is that the variability in PMF across individuals also meant that a peak frequency could not be identified for all participants in all experimental conditions. In our sample, the automated peak frequency calculation developed by Corcoran et al. (2018) could not identify a PMF for at least one condition in nearly one-third of our participants. Interestingly, this number is roughly in line with a previous report that included null findings for mu suppression effects during motor imagery and AO (Tangwiriyasakul et al., 2013). Thus, there may be fundamental differences in the stability of alpha-band activity across individuals that make measurements of the PMF more reliable in some individuals than others. More broadly, growing research suggests that contributions of alpha-band oscillations may be dissociable from underlying aperiodic (1/*f*-like) activity (Donoghue et al., 2020), which is ignored in many studies including our own. In some cases, accounting for aperiodic activity has been shown to remove or reduce the predictive power of alpha oscillations, such as for cognitive processing speed (Ouyang et al., 2020) and aging (Donoghue et al., 2020). On the other hand, separating periodic and aperiodic components of the EEG signal has been suggested to produce rich and reliable data for distinguishing ASD and neurotypical populations (Levin et al., 2020). Additionally, because these methods are data-driven and do not require *a priori* definitions of the frequency bands of interest, these approaches may be valuable both for detecting peaks outside of the predefined alpha range and for exploring a wider space of oscillatory brain rhythms.

In conclusion, our results provide new insights into how the sensorimotor mu rhythm varies across individuals within a neurotypical sample. Individual measurement in the peak oscillatory frequency of the mu rhythm is highly reliable, is not differentially driven by resting vs. task states, and shows good correlation across different tasks associated with mu suppression. As predicted, higher autism-spectrum traits were associated with slowing of the mu rhythm, extending previous developmental findings linking peak alpha-band frequency to ASD. Further research with larger samples, both in neurotypical and clinical populations, is warranted to further explore how individual peak oscillatory alpha-band frequencies vary as a function of autism-spectrum traits.

## Data availability statement

The raw data supporting the conclusions of this article will be made available by the authors, without undue reservation.

## Ethics statement

The studies involving human participants were reviewed and approved by the Claremont McKenna College Institutional Review Board. The patients/participants provided their written informed consent to participate in this study.

## Author contributions

AH, CR, and EM contributed to conception and design of the study. CS and AH collected the data, performed the statistical analysis, and wrote the first draft of the manuscript. All authors contributed to manuscript revision, read, and approved the submitted version.
